# IL-10 inhibition during immunization improves vaccine-induced protection against *Staphylococcus aureus* infection

**DOI:** 10.1172/jci.insight.178216

**Published:** 2024-05-28

**Authors:** Alanna M. Kelly, Karen N. McCarthy, Tracey J. Claxton, Simon R. Carlile, Eoin C. O’Brien, Emilio G. Vozza, Kingston H.G. Mills, Rachel M. McLoughlin

**Affiliations:** 1 Host-Pathogen Interactions Group and; 2 Immune Regulation Research Group, School of Biochemistry and Immunology, Trinity Biomedical Sciences Institute, Trinity College Dublin, Dublin, Ireland.

**Keywords:** Immunology, Vaccines, Bacterial infections, Bacterial vaccines, T cells

## Abstract

*Staphylococcus aureus* is a major human pathogen. An effective anti–*S*. *aureus* vaccine remains elusive as the correlates of protection are ill-defined. Targeting specific T cell populations is an important strategy for improving anti–*S*. *aureus* vaccine efficacy. Potential bottlenecks that remain are *S*. *aureus*–induced immunosuppression and the impact this might have on vaccine-induced immunity. *S*. *aureus* induces IL-10, which impedes effector T cell responses, facilitating persistence during both colonization and infection. Thus, it was hypothesized that transient targeting of IL-10 might represent an innovative way to improve vaccine efficacy. In this study, IL-10 expression was elevated in the nares of persistent carriers of *S*. *aureus*, and this was associated with reduced systemic *S*. *aureus*–specific Th1 responses. This suggests that systemic responses are remodeled because of commensal exposure to *S*. *aureus*, which negatively implicates vaccine function. To provide proof of concept that targeting immunosuppressive responses during immunization may be a useful approach to improve vaccine efficacy, we immunized mice with T cell–activating vaccines in combination with IL-10–neutralizing antibodies. Blocking IL-10 during vaccination enhanced effector T cell responses and improved bacterial clearance during subsequent systemic and subcutaneous infection. Taken together, these results reveal a potentially novel strategy for improving anti–*S*. *aureus* vaccine efficacy.

## Abstract 2

IL-10 inhibition during vaccination against* Staphylococcus aureus* improves vaccine efficacy and indicates that nasal colonization influences systemic T cell responses.

## Introduction

*S*. *aureus* is one of the leading causes of both community-acquired and hospital-acquired bacterial infections and is associated with more than 1 million deaths annually (1, 2). *S*. *aureus* is a major source of fatal bloodstream infections, where it carries a significant mortality risk (3). However, the most common manifestations of *S*. *aureus* infections are chronic and recurrent skin and soft tissue infections (SSTIs) (4). The treatment of staphylococcal infections continues to be increasingly challenging because of the rapid emergence of antimicrobial resistance (AMR), with *S*. *aureus* being the leading cause of AMR deaths in high-income countries (2, 5). Although infection prevention efforts have reduced the incidence of methicillin-resistant *S*. *aureus* infections in many countries, this has coincided with an increase in the rates of methicillin-sensitive infections (6, 7). Therefore, the health risk posed by *S*. *aureus* infection urgently necessitates the development of novel effective therapies that improve clinical outcomes by providing broad-spectrum protection, regardless of antimicrobial resistance patterns. The development of an efficacious, prophylactic anti–*S*. *aureus* vaccine is now more urgent than ever, as treatment options narrow further.

Despite significant efforts over the past 2 decades, there is still no effective vaccine that protects against *S*. *aureus* infection. It is now widely accepted that antibody-based vaccination strategies are not working and that effective *S*. *aureus* vaccines will need to induce protective cellular as well as humoral immunity (8–11). The benefit of vaccine-induced T cell responses is supported by post hoc analysis of the phase IIb/III study of the failed Merck vaccine candidate. This study demonstrated 100% mortality in vaccinated patients with undetectable IL-2 levels who subsequently developed *S*. *aureus* infection, suggesting that even in the presence of an antibody response, a protective immune response to *S*. *aureus* requires the generation of certain T cell subsets (12). This is consistent with evidence from murine models that Th1 and Th17 cells play a pivotal role in clearance of *S*. *aureus* (13–15). Consequently, effective vaccines against *S*. *aureus* will require appropriate adjuvants to drive Th1 and/or Th17 responses, which are necessary for protective immunity at different anatomical sites of infection (15–17).

There is an increasing appreciation of how the microbiota can influence vaccine-induced immune responses (18, 19), with an association between the composition of the infant microbiota and immune responses to vaccination reported in several clinical studies (20, 21). Gut microbiome species can enhance plasma cell activation through TLR5, and this directly correlated with the magnitude of the antibody response to the seasonal flu vaccine (22). In addition, the skin microbiome has been shown to influence the immune response to dermal vaccination with vaccinia virus (VACV) (23). The dermal microbiota provides an adjuvant-like stimulus during VACV vaccination, thereby enhancing the protective host immune response induced by the vaccine.

Conversely, the microbiota can also negatively influence vaccine efficacy. High abundance of *Enterobacteriales* and *Pseudomonadales* in the gut of Bangladeshi children were negatively associated with the function of tetanus toxoid and antituberculosis Bacille Calmette-Guerin (BCG) vaccines, resulting in lower vaccine-induced responses (24, 25). Additionally, the microbiota has been reported to inhibit BCG-induced protection against *Mycobacterium tuberculosis* infection by skewing vaccine-induced immunity in favor of suppressive Tregs (26). Preexisting immunity to mycobacterial antigens has also been linked to increased numbers of TGF-β–producing CD4^+^CD25^+^CD39^+^ Tregs and a reduced activation of Th17 responses following immunization (27). Taken together these data suggest that exposure to organisms with the capacity to drive immunosuppression can interfere with vaccine-induced effector responses, required for protection against the same and possibly other organisms.

There is a growing body of evidence that exposure to *S*. *aureus* may “imprint” the immune system in a suppressive manner. Consequently, an individual’s past exposure, particularly in the context of colonization, could affect the ability of vaccines to drive protective T cell responses during subsequent infection. During both nasal colonization and skin infection, *S*. *aureus* drives local production of the immunosuppressive cytokine IL-10 as a means of dampening effector T cell responses, thereby facilitating bacterial persistence (28, 29). Whether there is an immunosuppressive imprint induced by prior *S*. *aureus* exposure that could interfere with vaccine-induced T cell function remains to be established. If this is the case, inhibition of antiinflammatory immune responses during the vaccination might prove an effective means of enhancing vaccine efficacy.

The current study demonstrates that healthy adults who are persistently colonized with *S*. *aureus* exhibit an enhanced local IL-10 response in the nasal tissue as compared with noncolonized individuals, which is associated with dysregulated systemic *S*. *aureus* antigen-specific effector T cell responses, supporting the conjecture that prior exposure to *S*. *aureus* through nasal colonization could influence vaccine function. Studies in mice demonstrate that inhibition of IL-10 during immunization augments anti–*S*. *aureus* T cell responses, leading to enhanced protection against subsequent *S*. *aureus* infection. IL-10 inhibition increased vaccine efficacy of a CpG-based or a potentially novel TLR2 + stimulator of interferon genes (STING) agonist–based adjuvant, which drive strong Th1 and Th17 responses, respectively. Overall, this work reveals the critical role that bacterially induced immunosuppression could have on the effectiveness of anti–*S*. *aureus* vaccines and highlights a potential method to improve vaccine efficacy.

## Results

### IL-10 expression is upregulated in the nasal cavity of S. aureus persistently colonized individuals, and this is associated with altered S. aureus antigen-specific systemic T cell responses.

**Figure 1:**
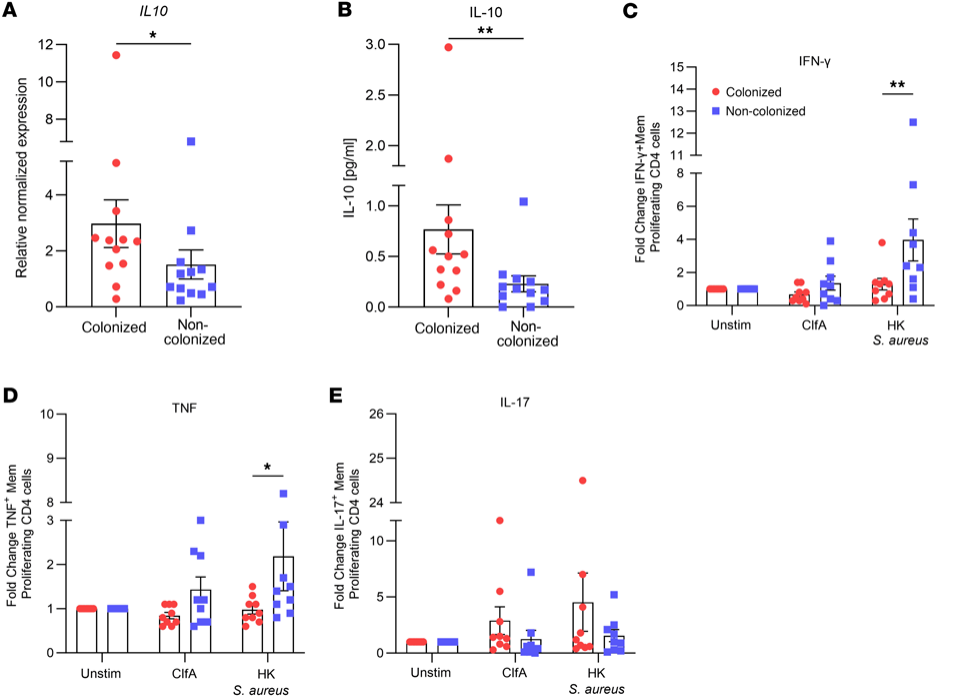
Persistent nasal colonization with *S*. *aureus* is associated with enhanced IL-10 responses locally within the nasal tissue and altered systemic T cell responses upon *S*. *aureus* reexposure. Persistently colonized individuals were identified as those who had 3 consecutive nasal swab cultures positive for *S*. *aureus* over a 6-week period. Individuals who tested negative for each swab culture were classified as “noncolonized.” Nasal mucosa was swabbed, and RNA was extracted. *IL10* gene expression levels were assessed using quantitative reverse transcription PCR (**A**). The mRNA values were expressed as mean relative expression ± SEM and compared with baseline IL-10 expression from noncolonized individuals after normalizing to β-actin RNA expression. (Experimental unit = 1 donor, *n* = 12/group.) Mucosal lining fluid (MLF) was collected using Nasosorption FX·i devices, and IL-10 concentration was measured using a V-plex multiplex ELISA (**B**). Results are expressed as mean protein expression ± SEM. (Experimental unit = 1 donor, *n* = 12/group.) Purified CD4^+^ T cells were carboxyfluorescein diacetate succinimidyl ester–labeled (CFSE-labeled) and cocultured with autologous irradiated antigen-presenting cells from a subgroup of persistently colonized or noncolonized individuals. Cells were stimulated with media alone, ClfA (0.88 μM), or heat-killed *S*. *aureus* Newman strain (1 μg/mL) for 8 days. The proportions of IFN-γ^+^ (**C**), TNF^+^ (**D**), and IL-17^+^ (**E**) proliferating memory CD45RO^+^CD4^+^ T cells were then assessed. Values are expressed as mean fold-change ± SEM. (Experimental unit = 1 donor, *n* = 9/group.) Statistical analysis was carried out by Mann-Whitney *U* test to analyze variances between groups for gene expression and ELISA data or 2-way ANOVA with Holm-Šídák posttest for flow cytometry data. *P ≤ 0.05; **P ≤ 0.01.

In a murine model of *S*. *aureus* nasal colonization, local induction of IL-10 by the bacterium creates an immunosuppressive state within the nasal cavity to facilitate persistence (28). *S*. *aureus*–induced IL-10 dampened local effector T cell responses, which led to impaired bacterial clearance. To establish if persistent colonization with *S*. *aureus* is similarly associated with the induction of local immunosuppressive responses in humans, nasal swabs were collected from a cohort of healthy adults (Supplemental Table 1; supplemental material available online with this article; https://doi.org/10.1172/jci.insight.178216DS1), who were defined as persistently colonized or noncolonized based on previously established criteria (30), for analysis of IL-10 at the gene and protein levels. Persistently colonized individuals had significantly higher expression of IL-10 mRNA in nasal mucosa compared with noncolonized individuals (Figure 1A). IL-10 protein concentrations in mucosal lining fluid were also significantly enhanced in the persistently colonized compared with noncolonized individuals (Figure 1B). While overall low, the levels detected were consistent with levels previously detected in nasal washes from healthy individuals (31–35). Low levels of expression of IL-17 and IL-22 were observed at both the gene and protein levels, with no difference detectable between persistently colonized and noncolonized individuals (Supplemental Figure 1).

To determine if there were any systemic consequences for this immunosuppressive phenotype associated with persistent *S*. *aureus* nasal colonization, peripheral blood mononuclear cells (PBMCs) were collected from a subset of persistently colonized and noncolonized individuals and cultured in vitro in the presence of heat-killed *S*. *aureus*, which served as a pool of *S*. *aureus* antigens, or the purified *S*. *aureus* antigen ClfA, to assess *S*. *aureus* antigen-specific memory T cell responses. After 8 days in culture, cytokine production by proliferating memory CD45RO^+^CD4^+^ T cells was assessed. Persistently colonized individuals had lower frequency of antigen-specific IFN-γ^+^ (Figure 1C) and TNF^+^ (Figure 1D) proliferating memory CD4^+^ cells, concomitant with elevated proportions of IL-17^+^ proliferating memory CD4^+^ T cells (Figure 1E). The data suggest that *S*. *aureus* nasal colonization is creating an immunosuppressive imprint, which has an inhibitory effect on *S*. *aureus* antigen-specific circulating Th1 cells. The associated nonsignificant increase in antigen-specific IL-17 responses may be a compensatory mechanism (36), evident in the in vitro system, whereby upon stimulation, the impaired IFN-γ response allows IL-17 to be more readily detected. Interestingly, other studies have similarly found reduced antigen-specific Th1 responses in noncarriers (37).

### IL-10 inhibition during immunization improves vaccine efficacy against systemic S. aureus infection.

**Figure 2:**
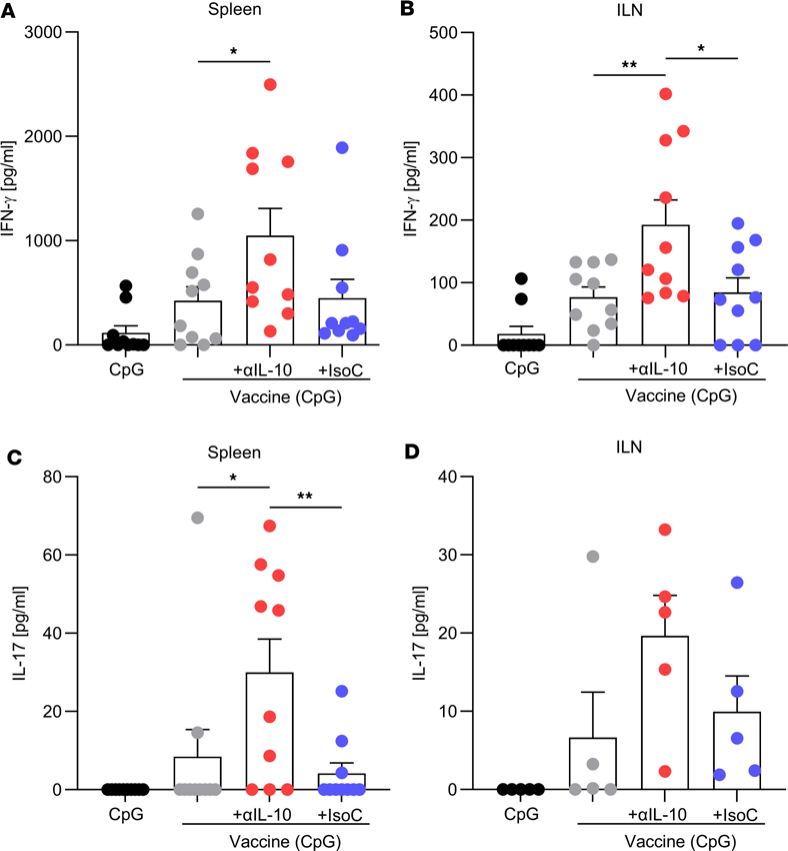
Immunization in the presence of anti–IL-10 increases ClfA-specific IFN-γ and IL-17 production by T cells of the spleen and ILNs. Mice were immunized with CpG (50 μg); vaccine only, consisting of CpG + ClfA (5 μg); vaccine + anti–IL-10 (150 μg); or vaccine + isotype control (150 μg) via s.c. injection on day 0, 14, and 28. On day 42 ILNs and spleen were removed, and ClfA-specific responses were assessed by ex vivo stimulation with media only or ClfA (5 μg/mL) for 72 hours. The levels of IFN-γ in the spleen (**A**) and ILNs (**B**), and IL-17 in the spleen (**C**) and ILNs (**D**), were determined by ELISA. ClfA-specific responses were determined by subtracting responses to media alone. Results are expressed as mean ± SEM. (Experimental unit = 1 mouse, *n* =5–10/group, total mice used 40, experiment was performed twice.) Statistical analysis was carried out by 1-way ANOVA with Tukey posttest. *P ≤ 0.05, **P ≤ 0.01.

Having demonstrated that colonization status impacts systemic *S*. *aureus* antigen-specific T cell responses in humans, potentially due to an immunosuppressive imprint conferred by persistent carriage of the bacterium, it was hypothesized that inhibition of immunosuppression during immunization might be an effective way to improve *S*. *aureus* vaccine efficacy. We used a murine model to provide proof of concept that this could be achieved. Previous work has shown that an experimental vaccine against *S*. *aureus*, comprising CpG (TLR9 agonist) with the *S*. *aureus* cell wall–anchored protein ClfA, effectively protected against *S*. *aureus* systemic infection through the induction of Th1 responses (15). To determine if blockade of IL-10 during immunization could improve the efficacy of this vaccine, groups of C57BL/6J WT mice were immunized s.c. with CpG (50 μg) alone, vaccine (comprising ClfA [5 μg] + CpG [50 μg]), vaccine + anti–IL-10 (150 μg), or vaccine + isotype control (150 μg) on days 0, 14, and 28. Cellular and humoral immune responses were assessed on day 42. Inguinal lymph nodes (ILNs) and spleen were removed, and ClfA-specific memory T cell responses were assessed. The production of IFN-γ (Figure 2, A and B) and IL-17 (Figure 2, C and D), in response to in vitro ClfA reexposure, was higher in cells isolated from mice immunized with the vaccine + anti–IL-10 compared with mice immunized with vaccine + isotype or vaccine alone. IL-17 responses were lower than those of IFN-γ, likely due to the Th1-skewing adjuvant CpG (38). Sera from mice immunized with the experimental vaccine effectively inhibited the binding of *S*. *aureus* to fibrinogen, verifying the presence of ClfA-neutralizing antibodies. However, the addition of the IL-10–blocking antibody during immunization had no significant effect on ClfA-specific antibody levels (Supplemental Figure 2).

**Figure 3:**
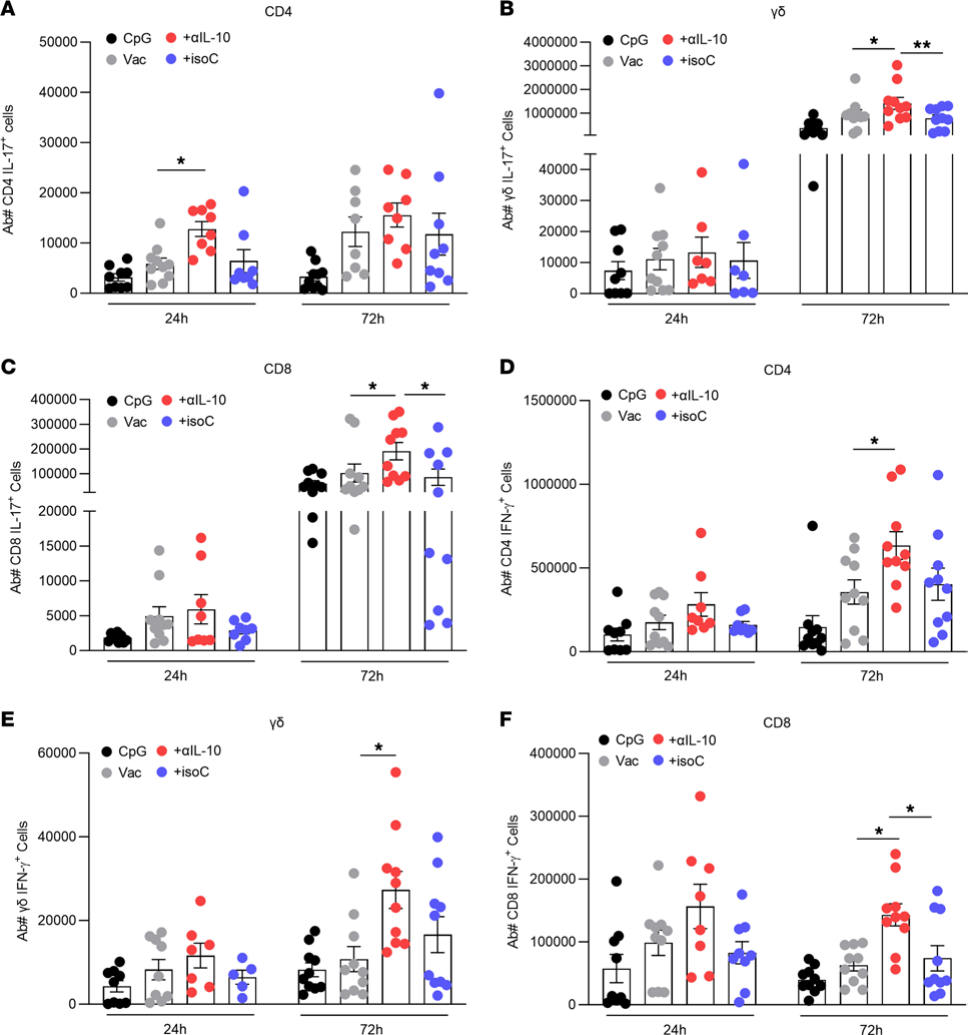
IL-10 inhibition during vaccination with a CpG-based *S*. *aureus* vaccine improves T cell immune responses during systemic infection. Mice were immunized with CpG (50 μg); vaccine only, consisting of CpG + ClfA (5 μg); vaccine + anti–IL-10 (150 μg); or vaccine + isotype control (150 μg) via s.c. injection on day 0, 14, and 28. On day 42 mice were challenged with *S*. *aureus* strain PS80 (5 × 10^8^ CFU) via i.p. injection. At 24 hours and 72 hours after infection, cells of the peritoneal cavity were isolated to assess the number of IL-17^+^ CD4^+^ (**A**), γδ (**B**), and CD8^+^ (**C**) T cells and the number of IFN-γ^+^ CD4^+^ (**D**), γδ (**E**), and CD8^+^ (**F**) T cells in the peritoneum by flow cytometry. Results are expressed as mean absolute number of cell type ± SEM. (Experimental unit = 1 mouse, *n* = 7–10/group total mice used; 80, experiment was performed twice.) Statistical analysis was carried out by 2-way ANOVA with Holm-Šídák posttest. *P ≤ 0.05, **P ≤ 0.01.

**Figure 4:**
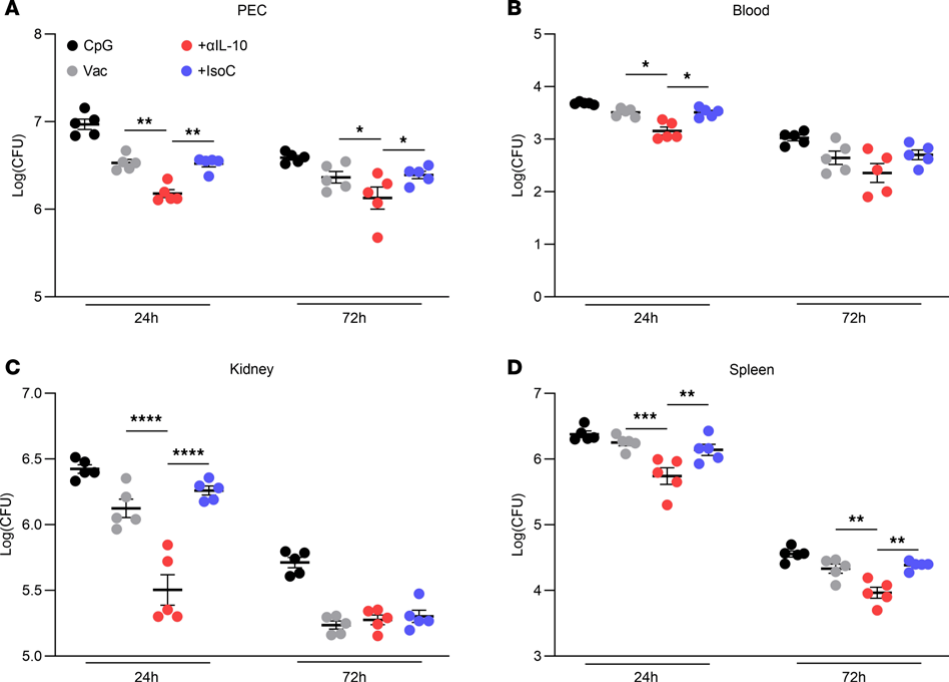
IL-10 inhibition during vaccination with a CpG-based *S*. *aureus* vaccine improves bacterial clearance during systemic infection. Mice were immunized with CpG (50 μg); vaccine only, consisting of CpG + ClfA (5 μg); vaccine + anti–IL-10 (150 μg); or vaccine + isotype control (150 μg) via s.c. injection on day 0, 14, and 28. On day 42 mice were challenged with *S*. *aureus* strain PS80 (5 × 10^8^ CFU) via i.p. injection. At 24 hours and 72 hours after infection bacterial burden was assessed in the peritoneal cavity (**A**), blood (**B**), kidney (**C**), and spleen (**D**). Results are expressed as Log(CFU) ± SEM. (Experimental unit = 1 mouse, *n* = 5/group, total mice used 40, experiment was performed once.) Statistical analysis was carried out by 2-way ANOVA with Holm-Šídák posttest. *P ≤ 0.05, **P ≤ 0.01, ***P ≤ 0.001, ****P ≤ 0.0001.

On day 42 after vaccination, mice were challenged i.p. with live *S*. *aureus* strain PS80 (5 × 10^8^ CFU). At 24 hours and 72 hours after infection, the cells infiltrating the peritoneal cavity were analyzed by flow cytometry. Cells were gated into IL-17^+^ and IFN-γ^+^ CD4^+^, CD8^+^, or γδ^+^ T cell subsets (Supplemental Figure 3). At 24 hours and 72 hours after infection, significant increases in the frequency of both IL-17–producing Th17 (Figure 3A) and IFN-γ–producing Th1 (Figure 3D) cells were observed in the peritoneal cavity of mice that were immunized with the vaccine + anti–IL-10 compared with control groups. Interestingly, immunization with vaccine + anti–IL-10 also led to significant increases in populations of IL-17–producing and IFN-γ–producing γδ^+^ T cells (Figure 3, B and E) and CD8^+^ cells (Figure 3, C and F). Importantly, these enhanced local T cell responses led to improved clearance of the infection. Significantly reduced levels of bacteria were detected at the site of infection in the peritoneal cavity (PEC) (Figure 4A) and in systemic organs in the mice that received vaccine + anti–IL-10 (Figure 4, B–D) compared with mice administered with the vaccine alone or vaccine + isotype control.

### IL-10 inhibition during vaccination improves vaccine efficacy against s.c. S. aureus infection.

**Figure 5:**
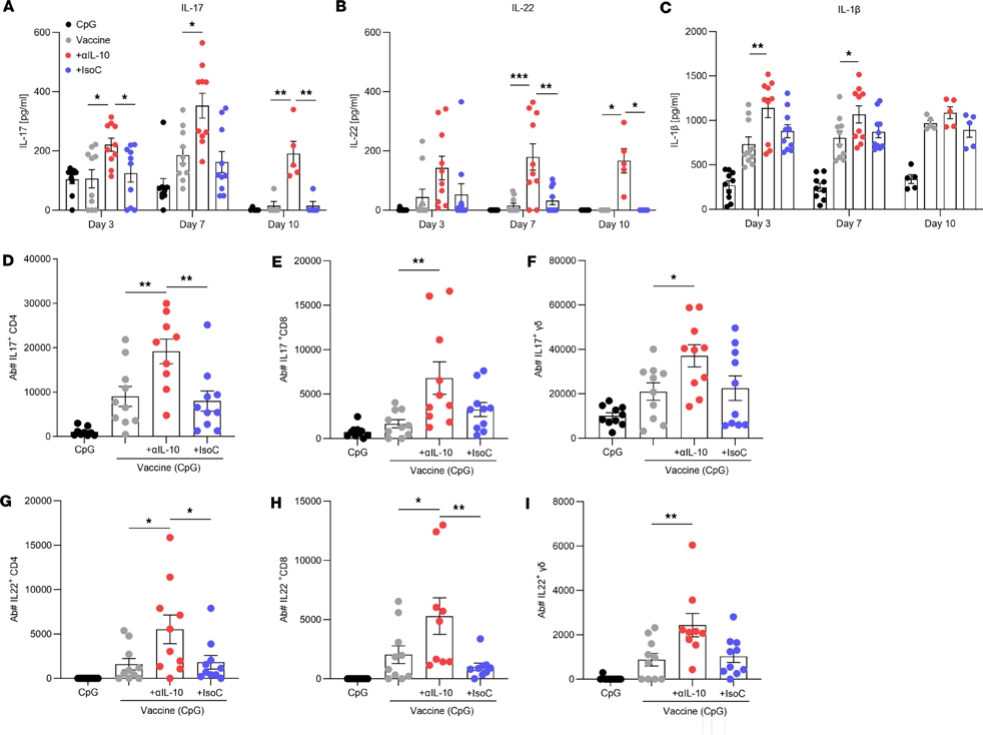
IL-10 inhibition during vaccination improves T cell immune responses during a subsequent *S*. *aureus* s.c. infection. Mice were immunized with CpG (50 μg); vaccine only, consisting of CpG + ClfA (5 μg); vaccine + anti–IL-10 (150 μg); or vaccine + isotype control (150 μg). All injections were via s.c. injection on day 0, 14, and 28. On day 42 mice were s.c. infected with *S*. *aureus* USA300 (LAC) (2 × 10^7^ CFU). On day 3, 7, and 10 after infection, an 8 mm skin punch biopsy was taken at the infection site and homogenized, and undiluted homogenate supernatants were used for IL-17 (**A**), IL-22 (**B**), and IL-1β (**C**) cytokine production analysis by ELISA. Results are expressed as mean protein expression ± SEM. (Experimental unit = 1 mouse, *n* = 5–10/group, total mice used 100, experiment was performed twice.) At 72 hours after infection cells of the abscess were isolated to assess the number of IL-17^+^ CD4^+^ (**D**), CD8^+^ (**E**), and γδ (**F**) T cells and the number of IL-22^+^ CD4^+^ (**G**), CD8^+^ (**H**), and γδ (**I**) T cell subtypes by flow cytometry. Results are expressed as mean absolute number of cell type ± SEM. (Experimental unit = 1 mouse, *n* = 9–10/group, total mice used 40, experiment was performed twice.) Statistical analysis was carried out by 1-way ANOVA with Tukey posttest for flow cytometry data or 2-way ANOVA with Holm-Šídák posttest for ELISA data. *P ≤ 0.05; **P ≤ 0.01, ***P ≤ 0.001.

**Figure 6:**
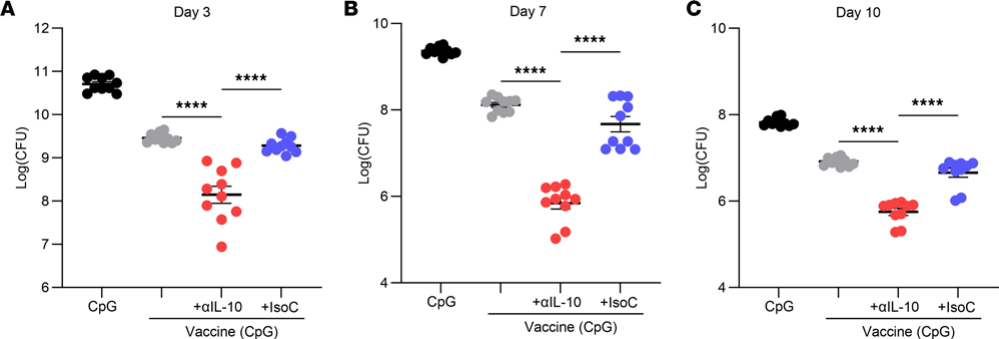
IL-10 inhibition during vaccination improves clearance of *S*. *aureus* during a subsequent s.c. infection. Mice were immunized with CpG (50 μg); vaccine only, consisting of CpG + ClfA (5 μg); vaccine + anti–IL-10 (150 μg); or vaccine + isotype control (150 μg). All injections were via s.c. injection on day 0, 14, and 28. On day 42 mice were s.c. infected with *S*. *aureus* USA300 (LAC) (2 × 10^7^ CFU). On day 3 (**A**), 7 (**B**), and 10 (**C**) after infection, an 8 mm skin punch biopsy was taken at the infection site and homogenized and bacterial burden was assessed. Results are expressed as Log(CFU) ± SEM. (Experimental unit = 1 mouse, *n* = 10/group, total mice used 120, experiment was performed twice.) Statistical analysis was carried out by 1-way ANOVA with Tukey posttest. ****P ≤ 0.0001.

Having demonstrated vaccine efficacy against systemic infection could be significantly improved by IL-10 inhibition, it was next investigated if this would also improve vaccine function in the context of a localized skin and soft tissue infection, where *S*. *aureus* has previously been shown to drive immunosuppressive IL-10 responses to promote persistence (29). Mice were immunized with CpG (50 μg), vaccine CpG + ClfA (5 μg), vaccine + anti–IL-10 (150 μg), or vaccine + isotype control (150 μg) via s.c. injection on day 0, 14, and 28. On day 42 mice were s.c. infected with *S*. *aureus* USA300 (LAC) (2 × 10^7^ CFU), as previously described (39–41). On day 3, 7, and 10 after infection the cutaneous tissue containing the skin abscess was collected using an 8 mm punch biopsy, and total tissue cytokine production was assessed. T cell–associated cytokines IL-17 (Figure 5A) and IL-22 (Figure 5B) were significantly increased in mice immunized with vaccine + anti–IL-10 when compared with control groups, and production was also sustained up to day 10 after infection. Flow cytometry analysis revealed that CD4^+^, CD8^+^, and γδ^+^ T cell subsets (Supplemental Figure 4) all contributed to both IL-17 (Figure 5, D–F) and IL-22 (Figure 5, G–I) production on day 3 after infection, and the inhibition of IL-10 significantly enhanced the production of these cytokines by all T cell subsets. Production of the innate cytokine IL-1β was also assessed (Figure 5C), as this cytokine has been shown to be important in the recruitment of neutrophils to the site of infection during *S*. *aureus* skin infection and for abscess formation (42). The presence of the anti–IL-10 antibody during vaccination significantly enhanced production of IL-1β in the skin compared with the control groups. Importantly, mice that received the IL-10–blocking antibody during immunization demonstrated significantly improved bacterial clearance compared with mice that received vaccine + isotype or the vaccine only at each time point (Figure 6, A–C).

The induction of IL-17 and IL-22 responses is known to be critically important for conferring protection against *S*. *aureus* SSTI (43). It was therefore investigated if the use of a novel adjuvant, LP1569 + cyclic-di-GMP (cGMP), which has previously been shown to be a potent IL-17–inducing adjuvant (44, 45), could similarly protect against *S*. *aureus* skin infection through induction of protective IL-17 responses (46). Furthermore, it has been shown that CpG itself can lead to the upregulation of IL-10 as well as inducing a Th1 type response (47); thus, the use of this novel Th17-inducing adjuvant may be more appropriate in the context of *S*. *aureus* SSTI. LP1569 is a synthetic TLR2 agonist based on the lipoprotein BP1569 from *B*. *pertussis*, while cGMP acts as a STING agonist (44, 45).

**Figure 7:**
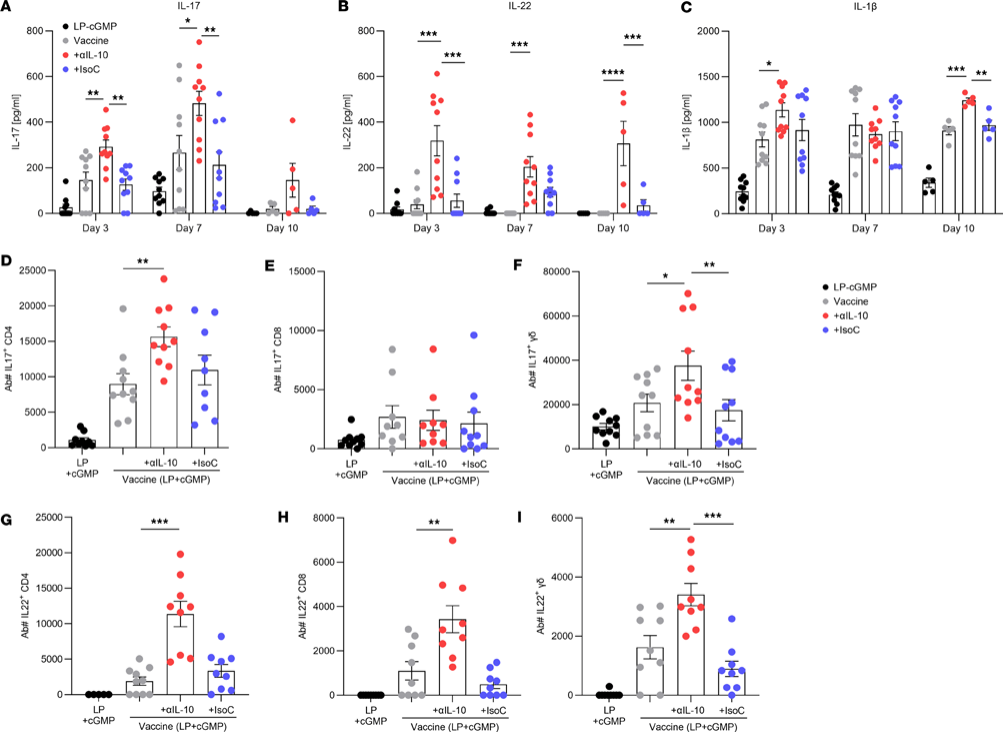
IL-10 inhibition during vaccination with a novel adjuvant improves T cell immune responses against a s.c. *S*. *aureus* infection. Mice were immunized with LP1569 (50 μg) + cGMP (10 μg); vaccine only, consisting of LP1569 + cGMP + ClfA (5 μg); vaccine + anti–IL-10 (150 μg); or vaccine + isotype control (150 μg). All injections were via s.c. injection on day 0, 14, and 28. On day 42 mice were s.c. infected with *S*. *aureus* USA300 (LAC) (2 × 10^7^ CFU). On day 3, 7, and 10 after infection, an 8 mm skin punch biopsy was taken at the infection site and homogenized, and undiluted homogenate supernatants were then used for IL-17 (**A**), IL-22 (**B**), and IL-1β (**C**) cytokine production analysis by ELISA. Results are expressed as mean protein expression ± SEM. (Experimental unit = 1 mouse, *n* = 5–10/group, total mice used 100, experiment was performed twice.) At 72 hours postinfection cells were isolated from the skin to assess the number of IL-17^+^ CD4^+^ (**D**), CD8^+^ (**E**), and γδ (**F**) T cells and the number of IL-22^+^ CD4^+^ (**G**), CD8^+^ (**H**), and γδ (**I**) T cells by flow cytometry. Results are expressed as mean absolute number of cell type ± SEM. (Experimental unit = 1 mouse, *n* = 9–10/group, total mice used 40, experiment was performed twice.) Statistical analysis was carried out by 1-way ANOVA with Tukey posttest for flow cytometry data or 2-way ANOVA with Holm-Šídák posttest. *P ≤ 0.05, **P ≤ 0.01, ***P ≤ 0.001, ****P ≤ 0.0001.

**Figure 8:**
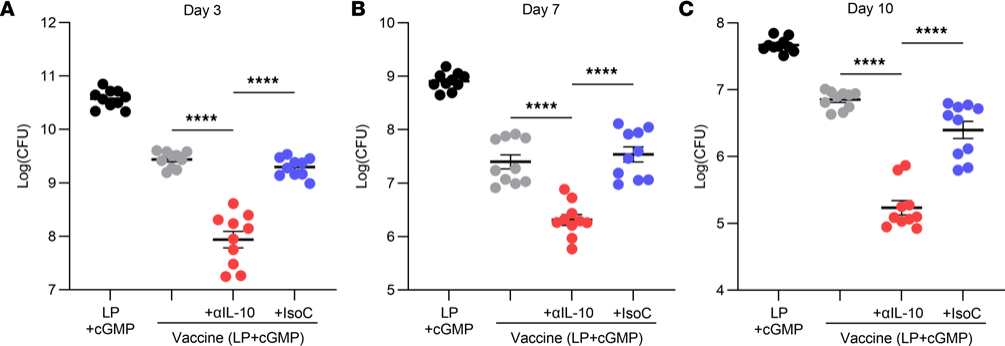
IL-10 inhibition during vaccination with a novel adjuvant improves clearance of *S*. *aureus* during a subsequent s.c. infection. Mice were immunized with LP1569 (50 μg) + cGMP (10 μg); vaccine only, consisting of LP1569 + cGMP + ClfA (5 μg); vaccine + anti–IL-10 (150 μg); or vaccine + isotype control (150 μg). All injections were via s.c. injection on day 0, 14, and 28. On day 42 mice were s.c. infected with *S*. *aureus* USA300 (LAC) (2 × 10^7^ CFU). On day 3 (**A**), 7 (**B**), and 10 (**C**) after infection, an 8 mm skin punch biopsy was taken at the infection site and homogenized and bacterial burden was assessed. Results are expressed as Log(CFU) ± SEM. (Experimental unit = 1 mouse, *n* = 10/group, total mice used 120). Statistical analysis was carried out by 1-way ANOVA with Tukey posttest. ****P ≤ 0.0001.

Mice were immunized with LP1569 (50 μg) + cGMP (10 μg) only; vaccine only, consisting of LP1569 (LP) + cGMP + ClfA (5 μg); vaccine + anti–IL-10 (150 μg); or vaccine + isotype control (150 μg), following the same regimen of injections and subsequent s.c. infection on day 42 with LAC (2 × 10^7^ CFU). Prior to infection, baseline levels of ClfA-specific memory T cell responses were assessed. The production of IFN-γ and IL-17 in response to in vitro ClfA reexposure was upregulated when anti–IL-10 was present during vaccination (Supplemental Figure 5). The adjuvant choice also impacted the type of ClfA-specific T cell cytokine responses induced. The LP + cGMP–based vaccine promoted induction of much higher concentrations of IL-17 (Supplemental Figure 5, C and D) compared with the CpG-based vaccine (Figure 2, C and D), with close to a 10-fold greater response observed in the ILN following immunization with the vaccine with LP + cGMP as the adjuvant. Following s.c. challenge with *S*. *aureus*, IL-17 and IL-22 production within the skin of the vaccine + anti–IL-10 group was significantly elevated compared with control groups (Figure 7, A and B), with higher levels of IL-17 and IL-22 produced by multiple T cell sources within the skin (Figure 7, D–I). Similarly to the CpG-based vaccine, the presence of the anti–IL-10 antibody during vaccination with the LP + cGMP–based vaccine significantly enhanced production of IL-1β in the skin compared with the control groups (Figure 7C). Overall, the enhanced T cell responses resulted in significantly improved bacterial clearance in the skin of vaccinated mice compared with the adjuvant alone (Figure 8, A–C), which was the first time to our knowledge this vaccine has been shown to have efficacy against *S*. *aureus* infection. When this vaccine was administered in combination with an anti–IL-10 blocking antibody, bacterial burden at the site of infection was further diminished as early as day 3 after infection (Figure 8, A–C).

**Figure 9:**
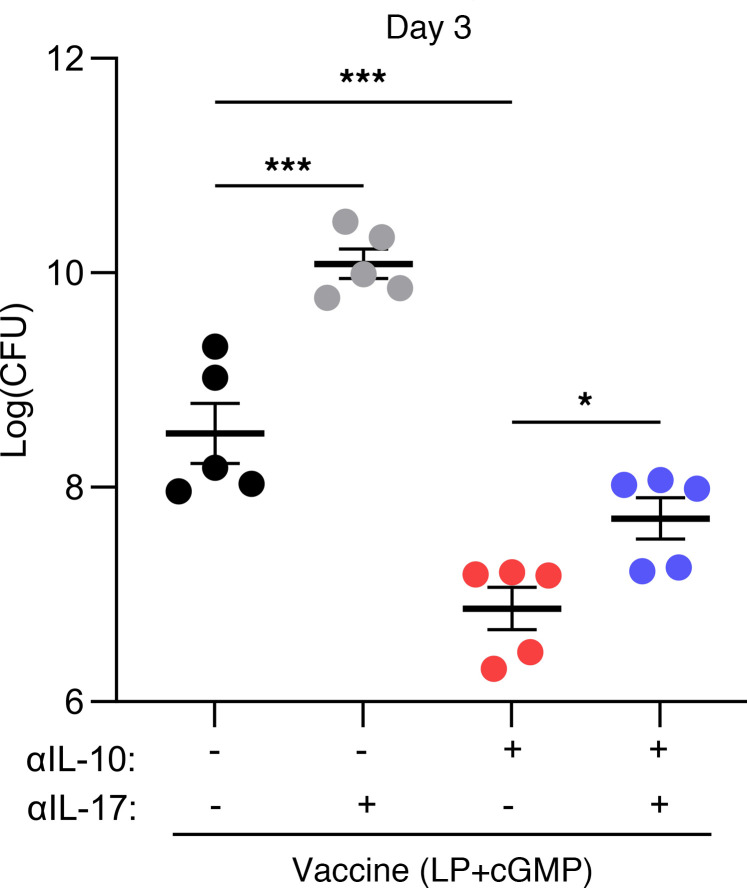
IL-17 blocking during s.c. *S*. *aureus* infection impedes bacterial clearance and reduces the protective effect of IL-10 inhibition during vaccination. Mice were immunized with LP1569 (50 μg) + cGMP (10 μg); vaccine only, consisting of LP1569 + cGMP + ClfA (5 μg); vaccine + anti–IL-10 (150 μg); or vaccine + isotype control (150 μg). All injections were via s.c. injection on day 0, 14, and 28. On day 42 mice were s.c. administered anti–IL-17 (50 μg) or isotype control (50 μg) alongside *S*. *aureus* USA300 (LAC) (2 × 10^7^ CFU) and again at 24 hours after infection. On day 3 after infection an 8 mm skin punch biopsy was taken at the infection site and homogenized and bacterial burden was assessed. Results are expressed as Log(CFU) ± SEM. (Experimental unit = 1 mouse, *n* = 5/group, total mice used = 20.) Statistical analysis was carried out by 1-way ANOVA with Tukey posttest. *P ≤ 0.05, ***P ≤ 0.001.

To demonstrate that enhanced T cell responses after vaccination with IL-10 inhibition were responsible for the improved bacterial clearance, IL-17 was blocked during s.c. infection (Supplemental Figure 6). The neutralization of IL-17 led to significantly increased bacterial burden in the skin in both the vaccine group + isotype and in the vaccine + anti–IL-10 group (Figure 9), showing that protection conferred by this vaccine is IL-17 dependent. Inhibition of this cytokine removed the beneficial impact of IL-10 blockade on vaccine function. Once IL-17 was blocked, levels of bacterial burden in the vaccine + anti–IL-10 group returned almost to those observed in the vaccine + isotype–only group, with no significance between groups, showing that IL-17–derived protection is critical. The small differences between the vaccine + isotype and vaccine + anti–IL-10 + anti–IL-17 may be due to other T cell–derived cytokines, such as IL-22 and/or a contribution of myeloid-induced protection.

Overall, these results indicate that inhibiting IL-10 production during vaccination improves vaccine efficacy against subsequent s.c. *S*. *aureus* infection, due to enhanced T cell responses at the site of infection in combination with enhanced IL-1β production, both of which are central to effective bacterial clearance.

## Discussion

Many decades of research have attempted to design an efficacious vaccine against *S*. *aureus* but to no avail. This study reveals a potential bottleneck that may need to be circumvented to realize an effective vaccine. Recently, it has been reported that vaccine interference impacts the generation of vaccine-induced protective anti–*S*. *aureus* antibody responses (48, 49). Prior *S*. *aureus* infection appears to condition the host immune response to produce nonprotective antibodies against *S*. *aureus* following immunization with the alum-based IsdB vaccine (48). It is possible that prior immune exposure to *S*. *aureus* could also impact vaccine-induced protective effector T cell responses, in particular, if memory T cells are preprogrammed to respond to *S*. *aureus* exposure in an antiinflammatory manner. Here, we demonstrate that *S*. *aureus* colonization drives the production of IL-10 within the nasal cavity of healthy adults, likely as a means of facilitating bacterial persistence. Importantly, this is associated with altered circulating memory T cell responses upon exposure to *S*. *aureus* antigens. This supports the hypothesis that prior exposure to *S*. *aureus*, as a consequence of colonization, may represent a significant barrier to the induction of protective T cell responses during vaccination. Using preclinical models, we demonstrate for the first time to our knowledge that the transient inhibition of IL-10 during immunization improves vaccine-induced T cell responses that lead to more effective clearance of infection. Importantly, this approach is most effective when the vaccine is administered with adjuvants that drive antigen-specific Th1/Th17 responses. Together the data support the conjecture that preprogrammed suppressive immune responses may contribute to the failure of current *S*. *aureus* vaccines and suggest that dampening IL-10 during vaccination could be a novel method of reprogramming host T cells to respond to *S*. *aureus* in a more protective manner.

Studies in murine models have demonstrated that expression of IL-27 and IL-10 are significantly enhanced in the nasal mucosa during *S*. *aureus* colonization, and these immunosuppressive cytokines directly impair local T cell responses, leading to inefficient bacterial clearance from the nasal cavity (28). This study demonstrates that humans who are persistently colonized with *S*. *aureus* display a similar immunosuppressive environment within the nasal mucosa, expressing significantly elevated levels of IL-10 production within the nasal cavity compared with noncolonized individuals. This likely suppresses local effector T cell cytokine production that would normally be induced in the tissue in response to the presence of *S*. *aureus*. Furthermore, this phenotype appears to be linked to impaired *S*. *aureus* antigen-specific circulating T cell responses in persistent carriers. The proportions of proliferating memory CD4^+^ T cells producing IFN-γ and TNF in response to *S*. *aureus* antigen stimulation were significantly reduced in persistently colonized individuals when compared with memory T cells from noncolonized individuals. Previous studies have also found enhanced antigen-specific Th1 responses in noncarriers (37) and have suggested that a high Th1-to-Th17 mRNA ratio reduced the likelihood of persistent *S*. *aureus* nasal colonization (50). Consistent with these results, we observed a skewing toward enhanced systemic IL-17 responses in persistently colonized individuals. Together, these results validate that *S*. *aureus* nasal colonization leads to a dysregulation in systemic memory T cell responses, in particular suppressing potentially protective Th1 cells. This could have significant consequences for vaccines designed to induce effective antigen-specific T cell responses.

We have previously demonstrated that the absence of IL-10 during *S*. *aureus* nasal colonization promotes effective bacterial clearance through enhanced effector CD4^+^, CD8^+^, and γδ^+^ T cell responses (28). Similarly, it has been shown that IL-10^–/–^ mice have lower bacterial burden at the site of infection during *S*. *aureus* skin infection because of enhanced effector γδ^+^ T cell migration into the skin abscess and increased IL-17 and IFN-γ secretion by γδ^+^ and CD4^+^ T cells, respectively, compared with WT mice (29). Here, we demonstrate *S*. *aureus* vaccine efficacy can be improved by transient inhibition of IL-10 at the time of immunization. For the first time to our knowledge, we show that blockade of IL-10 during vaccination can improve protection against both systemic and local skin infection with *S*. *aureus*. Blocking IL-10 during vaccination enhanced the frequency of IFN-γ^+^, IL-17^+^, and IL-22^+^ T cell subsets at the site of infection, which are critical for protection against *S*. *aureus* (15, 17, 51). These enhanced T cell responses also significantly improved the rate of bacterial clearance, with significant reductions in bacterial burden already seen at day 3 after infection. The inhibition of IL-17 during s.c. infection demonstrates that T cell–mediated responses are critical for the enhanced protection observed when IL-10 neutralization occurs during vaccination and once again highlights the crucial role of T cells in the protective response needed against *S*. *aureus* infection. There was little to no improvement in neutralizing antibody responses when IL-10 blocking occurred alongside vaccine administration. However, antibodies alone have been shown to be insufficient at eliciting an efficacious and long-term protective response in human studies, with all vaccines focusing on inducing a potent humoral response alone failing to significantly increase protection against infection (8, 11, 52). Driving conventional T cell responses has, however, become a focus for vaccine design because of the recognition of their importance in the effective clearance of *S*. *aureus*. However, “alternative” T cells such as γδ T cells may be a novel cellular target for successful next-generation *S*. *aureus* vaccines (10). Our data reiterate the importance of these innate-like cells, with γδ T cells contributing to the enhanced effector cytokine production seen in both the systemic and s.c. infection setting and IL-10 blockade also resulting in improved cytokine responses from these cells.

The lab-designed CpG-based vaccine alone worked well and led to improved clearance compared with the control group as has been observed previously (15, 40). Further enhancement in clearance because of IL-10 inhibition highlights just how potent a method of vaccine modulation it is. IL-10 inhibition was also shown to improve the efficacy of a novel TLR2 agonist/STING agonist–based vaccine that had never before been used against *S*. *aureus* infection (44). This adjuvant has previously been shown to be a potent Th17-skewing adjuvant that works well in the mucosal setting (45). The use of the LP1569-cGMP adjuvant led to an increase in IL-17–producing, *S*. *aureus*–specific, T cell–associated responses, with levels further enhanced when IL-10 was inhibited during immunization. The use of this adjuvant preferentially drove an IL-17 response as compared with when the CpG adjuvant had been used. The cGMP and LP1569 act synergistically to induce the production of IFN-γ, IL-12, and IL-23, the latter of which leads to the activation and expansion of Th17 cells (53). The efficacy of this adjuvant has only been shown in the context of *Bordetella pertussis* infection to date (45), where Th17 responses are critical (54, 55). The adjuvants’ ability to promote a strong Th17 response may make it a more suitable adjuvant candidate than CpG, particularly for targeting *S*. *aureus* skin infection, where IL-17 and IL-22 are thought to be important correlates of immune protection (17, 51).

Interestingly, the inhibition of IL-10 during the vaccination process also led to a substantial increase in production of IL-1β within the skin abscess. This was true for both the CpG-based and LP1569-based vaccines. This cytokine is important in the protective immune response against *S*. *aureus* s.c. infection, leading to neutrophil recruitment and the induction of antimicrobial peptides that aid in bacterial clearance (56, 57). It is possible that the enhanced IL-17^+^ T cell responses may be promoting this increase in IL-1β as IL-17 production by Vγ6^+^ γδ T cells has previously been shown to induce IL-1β and TNF production during *S*. *aureus* skin infection (58). IL-10 has also been shown to directly regulate IL-1β production in a number of ways, such as reducing pro–IL-1β levels through promotion of STAT3 activity, suppressing the expression of genes associated with the NLRP3 inflammasome and even the shedding of the IL-1R (59–61). The reduced IL-10 levels could be allowing amplified NLRP3 activation and IL-1β production during *S*. *aureus* skin infection. Further studies are needed to elucidate the method in which IL-10 is leading to increased IL-1β production.

IL-10 inhibition during vaccination has previously been shown to successfully improve the efficacy of DC-based cancer therapeutic vaccines (62, 63). The blockade of IL-10 can also improve vaccine-induced antiviral immune responses against lymphocytic choriomeningitis virus infection. The treatment of mice with an anti–IL-10R antibody in conjunction with a DNA vaccine increased the frequency and number of IFN-γ–producing CD8^+^ T cells compared with mice treated with the vaccine alone, leading to enhanced clearance of persistent viral replication (64). Similarly, inhibition of IL-10 also improved BCG vaccination results by enhancing protective Th1 and Th17 responses during a subsequent *M*. *tuberculosis* challenge (65). Murine studies have also shown IL-10 blockade after infection can improve memory Th17 responses against *S*. *aureus* infection (66). This current study underscores the paramount importance for human vaccination studies to take into consideration an individual’s colonization status. The targeting of immunoregulatory mediators, such as suppressive cytokines and regulatory cells, induced as a consequence of commensal exposure to a particular organism, may be a novel means of improving vaccine efficacy against opportunistic pathogens such as *S*. *aureus* (67). The transient blockade of IL-10 during the vaccination process is an effective way to promote stronger pro-inflammatory responses during a subsequent infection, without completely removing the ability of immune cells to produce IL-10 to resolve pro-inflammatory responses after the infection has been cleared.

There is a growing area of research using checkpoint inhibition and the transient depletion of Tregs to improve vaccine efficacy in antitumor vaccines (68, 69), something, which in theory, could also be applied to novel bacterial vaccines. Future work, however, will necessitate an increased understanding of the immunosuppressive phenotypes induced as a consequence of *S*. *aureus* colonization and in particular to identify the cellular source of IL-10, which is influenced by *S*. *aureus* colonization in humans. Previous studies have indicated that innate cell populations are important IL-10 producers during *S*. *aureus* exposure, which can suppress adaptive immune responses (70, 71). *S*. *aureus* colonization occurs in early life (72); thus, it is likely that an immunosuppressive imprint is present throughout life. However, blockade of IL-10 during immunization provides a mechanism to overcome these potential effects of this immunosuppression, regardless of whether the immunosuppressive phenotype is the cause or the consequence of *S*. *aureus* colonization. While the nasal cavity is the primary ecological niche of *S*. *aureus* from which the bacterium can go on to colonize extranasally (73, 74), colonization at other anatomical sites does occur (75, 76). Some studies have documented intestinal colonization occurring alone (77); it would therefore be interesting to investigate if intestinal carriage of *S*. *aureus* can induce similar immunosuppressive effects.

Overall, this study supports the premise that colonization status has a direct impact upon systemic *S*. *aureus*–specific memory T cell responses, which could be hindering vaccine function due to the promotion of an immunosuppressive state. We provide, for the first time to our knowledge, important insight into the critical role IL-10 plays in impeding anti–*S*. *aureus* vaccine efficacy by dampening vaccine-induced protective T cell responses. We propose a method of improving vaccine efficacy by inhibiting IL-10 production during the vaccination, which leads to enhanced antigen-specific T cell responses and improved bacterial clearance in both the systemic and s.c. infection settings. Importantly, these studies also reiterate the importance of adjuvant choice in vaccine design, in particular when targeting infection at different anatomical sites. The use of a novel TLR2/STING agonist adjuvant was shown to be particularly effective for targeting *S*. *aureus* skin infection and, furthermore, can be improved by the inhibition of IL-10. Taken together, these observations suggest *S*. *aureus*–induced immunosuppression must be contemplated for future vaccine design, and in particular, significant consideration should be given to the impact that colonization might have on vaccine function. The temporary cessation of the immunosuppressive response during vaccination may prove beneficial for next-generation vaccines against *S*. *aureus* that are attempting to generate potent effector T cell responses.

## Methods

### Sex as a biological variable.

Our study examined male and female animals, and similar findings are reported for both sexes. For human studies both men and women were involved (specified in Supplemental Table 1).

### Mice.

C57BL/6J wild-type mice were bred in-house at the Trinity College Dublin Comparative Medicine Unit. In all experiments inclusion criteria were healthy animals (male and female) aged 6–12 weeks. There were no exclusions. Experiments were typically carried out 2 times with 5 mice per group to establish the reproducibility of the results and to accommodate processing and analysis of material. To reduce bias, mice in all experiments were matched for sex and age. For each experiment, mice were allocated to their treatment group by randomization within blocks (nuisance variables: sex, cage location). Groups were allocated by the person performing the experiment. Relevant Animal Research: Reporting of In Vivo Experiments guidelines were followed.

### Bacteria.

*S*. *aureus* strains PS80, USA300 (LAC), and Newman have been previously described (78–80). Strains were grown from frozen stocks on tryptic soy agar (TSA) at 37°C for 18 hours. All bacterial suspensions were prepared in sterile PBS and concentrations measured at an optical density of 600 nm. CFU were verified by plating serial dilutions of each inoculum onto TSA.

Bacteria were heat-inactivated for the human assays, as previously described (15). Bacterial suspensions were inactivated at 90°C for 45 minutes and then washed to remove secreted proteins. Protein concentration was assessed and normalized using Pierce BCA Protein Assay Kit (Thermo Fisher Scientific).

### Protein purification.

Recombinant ClfA N123 (amino acids 40 to 559) protein (81) was purified from *E*. *coli* by Ni^2+^ affinity chromatography as previously described (82). Endotoxin was removed from the protein using 5 mL Pierce High-Capacity Endotoxin Removal Resin spin columns (Thermo Fisher Scientific).

### Murine immunization model.

Naive C57BL/6J mice were vaccinated via s.c. injection with 100 μL of adjuvant (CpG 50 μg from Hycult Biotech or LP1569 50 μg, + cGMP 10 μg from InvivoGen, adjuvant + ClfA 5 μg [i.e., vaccine], vaccine + blocking antibody [anti–IL-10 150 μg, 2BScientific], or vaccine + isotype control [IgG1 150 μg, 2BScientific]) on day 0, 14, and 28. Prior to challenge, on day 42, ILNs and spleens were collected for antigen recall responses. On day 42, mice were challenged with an *S*. *aureus* systemic or s.c. infection.

In some experiments mice were also administered with anti–IL-17 (50 μg, 2BScientific) or isotype control (IgG1 50 μg, 2BScientific) alongside *S*. *aureus* on the day of s.c. challenge and at 24 hours after infection.

### Murine systemic infection and s.c. abscess models.

For the systemic infection model, mice were infected by intraperitoneal injection of 5 × 10^8^ CFU of *S*. *aureus* PS80 in 100 μL PBS as previously described (15). Mice were euthanized at specific time points after infection, and the peritoneum was lavaged with 3 mL PBS for T cell cytokine analysis by flow cytometry. Blood and organs were isolated to assess bacterial burden.

For the s.c. abscess model, the dorsal area of mice was shaved and injected s.c. with LAC (2 × 10^7^ CFU) in 50 μL of sterile PBS as previously described (29, 41). To assess bacterial burden levels, 8 mm punch biopsies of lesioned skin were taken at day 3, 7, and 10 after infections. Tissue was homogenized in sterile PBS, and total bacterial burden was determined by plating out serial dilutions on TSA. The supernatants were assessed for cytokine production analysis by ELISA.

For flow cytometry analysis, cells were isolated from abscess tissue using a digestion cocktail consisting of collagenase XI (1 mg/mL MilliporeSigma), hyaluronidase (0.5 mg/mL MilliporeSigma), and DNase I (0.1 mg/mL MilliporeSigma) for 30 minutes at 37°C in a shaking incubator, then filtered through a 40 μm nylon Falcon cell strainer as previously described (28). Cells were rested in complete RPMI media supplemented with l-glutamine (MilliporeSigma), penicillin-streptomycin (MilliporeSigma), and 10% FBS (MilliporeSigma) for 30 minutes in a 37°C, 5% CO_2_, incubator.

### ELISA.

ELISAs for IL-17, IL-22, IFN-γ, and IL-1β (R&D DuoSet; R&D Systems) were performed on antigen recall baseline cell culture supernatants or supernatants of skin homogenates as per the manufacturer’s instruction.

Cellular immune responses to immunization were measured in ILNs and spleens of vaccinated mice. Cells were isolated by tissue disruption and plated at 5 × 10^5^/well in a 96-well plate. Cells were stimulated with a negative control of medium alone or purified ClfA (5 μg/mL) or a positive control of anti-CD3 (1 μg/mL, clone 17A2, Invitrogen) + PMA (10 ng/mL, Merck). After 72 hours’ incubation at 37°C and 5% CO_2_, the cell supernatants were collected for cytokine analysis by ELISA.

### Flow cytometry for in vivo models.

Isolated PEC and skin cells were incubated with PMA (50 ng/mL), ionomycin (500 ng/mL, Merck), and Brefeldin A (5 μg/mL, Cayman Chemical) for 4 hours at 37°C. Cells were then washed in PBS and stained with FixViability Dye eFluor506, followed by incubation in Fc block (αCD16/CD32) (Thermo Fisher Scientific) before extracellular surface staining with fluorochrome-conjugated antibodies (Invitrogen unless stated) against CD45 (clone: 30-F11), CD3 (clone 145-2C11), CD4 (clone GK1.5), CD8 (clone 53–6.7), and γδ TCR (clone GL3). Cells were fixed and permeabilized, followed by intracellular staining with fluorochrome-conjugated antibodies against IL-17 (BioLegend; clone TC11-18H10.1) and IL-22 (clone 1H8PWSR). Fluorescence minus one (FMO) samples were used as controls. Flow cytometric data were acquired with a BD LSR Fortessa or Cytek Aurora and analyzed using FlowJo software (Tree Star, Inc).

### Identification of S. aureus persistent carriers and noncarriers.

A human *S*. *aureus* colonization cohort was established in which healthy staff and students at Trinity College Dublin were invited to participate. Anterior nasal swabs were collected, streaked on Columbia Agar and Mannitol Salt Agar (both supplied by Microbiology Department, Trinity College Dublin), and incubated for 18 hours at 37°C. A minimum of 3 anterior nasal swabs were taken, at least 2 weeks apart, to establish whether bacterial carriage was persistent (3/3 positive) or transient (1–2/3 positive) or whether the individual was a noncarrier (3/3 negative). A subset of individuals in both the noncolonized and persistently colonized groups underwent venous blood sampling, and all participants underwent mid-turbinate nasal mucosal cell sampling and nasal mucosal lining fluid collection.

### RNA extraction, cDNA synthesis, and quantitative PCR.

Two mid-turbinate flocked nylon nasal swabs were collected per participant. RNA was extracted from nasal mucosal samples using QIAGEN miRNeasy kit as per manufacturer’s instructions. The RNA concentration of each sample was determined using a NanoDrop spectrophotometer (Applied Bioscience) and concentration was equalized across samples. RNA samples were reverse-transcribed into cDNA using the Applied Bioscience High-Capacity cDNA Reverse Transcription Kit. mRNA was quantified using quantitative reverse transcription PCR on a CFX96 Touch Real-Time pCR Detection System (Bio-Rad) using iTaq Sybr Green Supermix (Bio-Rad) according to the manufacturer’s recommendations. Primer pairs are listed in Supplemental Table 2. The 2^-ΔΔCt^ method was used to calculate relative changes in gene expression.

### MLF ELISA.

MLF was collected using Nasosorption FX·i devices with a synthetic absorptive matrix. After collection samples were immediately placed at –80°C. The MLF was extracted and then diluted 1:2, and cytokine concentration was measured using a V-plex multiplex ELISA kit (Meso Scale Diagnostics) according to the manufacturer’s instructions.

### Isolation and stimulation of human T cells.

PBMCs were isolated using Lymphoprep gradient (Axis-Shield). CD4^+^ T cells were purified by MACS using the CD4^+^ T cell isolation kit (Miltenyi Biotec) and labeled with CFSE for proliferation analysis as previously described (15, 40, 83). PBMCs were γ-irradiated at 40 Gy with a 137 Cs source (GammaCell 3000, Best Theratronics). CFSE-labeled CD4^+^ cells (1 × 10^5^) were cocultured with irradiated PBMCs (1 × 10^5^) with complete RPMI only (negative control), purified ClfA (4.4 μM/mL), or heat-killed *S*. *aureus* strain Newman (1 μg/mL ~ 1 × 10^7^ CFU/mL). On day 8 T cell proliferation was assessed by CFSE staining, and memory cells were identified as those expressing CD4 (clone RPA-T4) and CD45RO (clone UCHL1). Cells were fixed and permeabilized, followed by intracellular staining with fluorochrome-conjugated antibodies against IL-17 (clone 201JS09), IFN-γ (clone 4S.B3), and TNF (clone Mab11). FMO samples were used as controls. Flow cytometric data were acquired with a BD LSR Fortessa or Cytek Aurora and analyzed using FlowJo software.

### Statistics.

Statistical analyses were performed using GraphPad Prism 9 software. For murine studies, differences between groups were analyzed using 1-way ANOVA with a Tukey comparison posttest or 2-way ANOVA with a Holm-Šidák correction posttest where appropriate. A *P* ≤ 0.05 was considered significant. For human studies, differences between groups were analyzed using Mann-Whitney *U* test or 2-way ANOVA with a Holm-Šidák correction posttest where appropriate. A *P* ≤ 0.05 was considered significant.

### Study approval.

All animal experiments were conducted in accordance with the recommendations and guidelines of the health product regulatory authority, the competent authority in Ireland, and in accordance with protocols approved by Trinity College Dublin Animal Research Ethics Committee. Euthanasia was by CO_2_ inhalation. Ethical approval for the human colonization study was granted by the Faculty of Health Sciences Ethics Committee, Trinity College Dublin. All participants gave written informed consent prior to participation and were allowed withdraw at any stage of the study.

### Data availability.

All relevant data are within the manuscript and its supplement. Values for all data points in graphs are reported in the Supporting Data Values file.

## Author contributions

AMK, KNM, KHGM, and RMM were responsible for conceptualization. AMK and RMM were responsible for formal analysis. KHGM and RMM were responsible for funding acquisition. AMK, KNM, TJC, SRC, ECO, and EGV were responsible for conducting experiments. AMK, KNM, TJC, and SRC were responsible for analyzing data. RMM was responsible for project administration and supervision. AMK was responsible for writing the original draft. AMK, SRC, ECO, EGV, KHGM, and RMM were responsible for review and editing of the manuscript.

## Supplementary Material

Supplemental data

Supporting data values

## Version 1 07/08/2024

Electronic publication
