# Supplementary material for: IL-10 inhibition during immunization improves vaccine-induced protection against Staphylococcus aureus infection
Source: JCI Insight. 2024 May 28;9(13):e178216. doi: 10.1172/jci.insight.178216 (PMC11383370; doi:10.1172/jci.insight.178216)
Supplement: Supplemental data [file jciinsight-9-178216-s162.pdf]

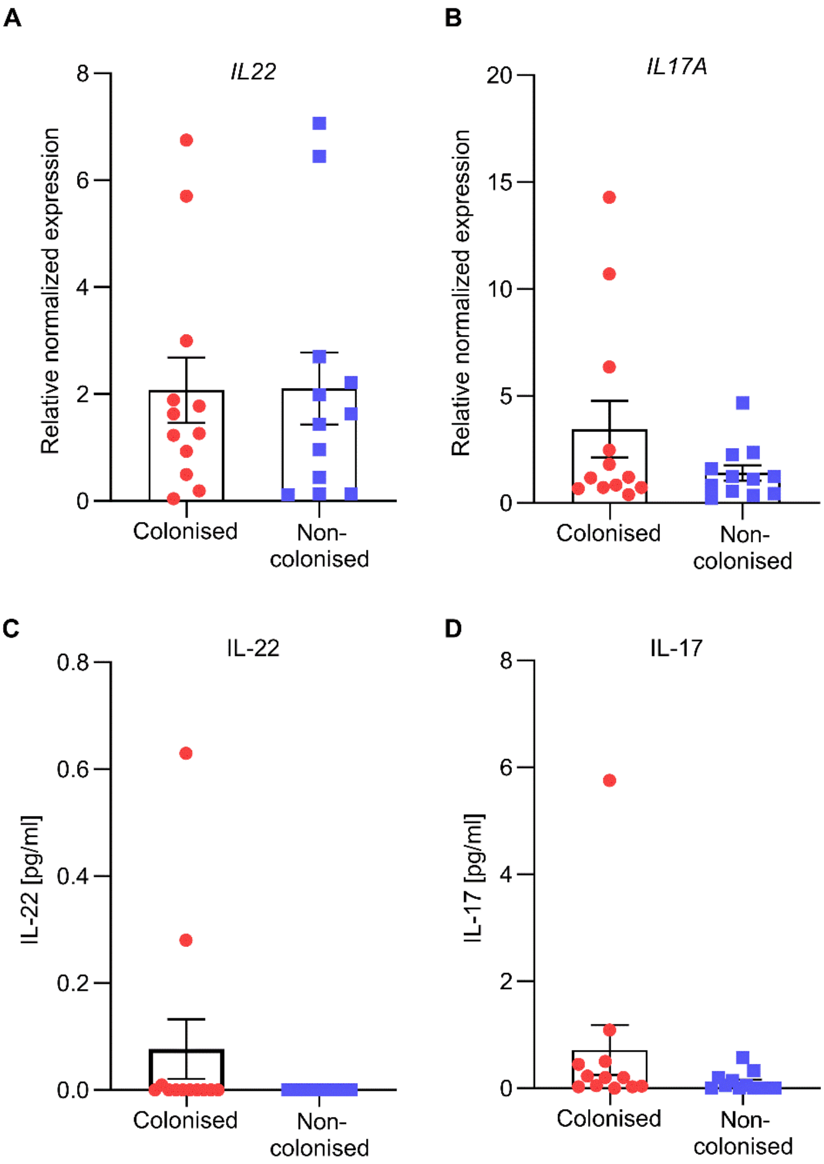

Supplemental Figure 1. Persistent colonisation with *S. aureus* is not associated with significantly enhanced IL-22 or IL-17 responses locally within the nasal tissue

Persistently colonised individuals were identified as those that had three consecutive nasal swab cultures positive for *S. aureus* over a six-week period. Individuals that tested negative at each swab culture, were classified as “non-colonised”. Nasal mucosa was swabbed, and RNA extracted. IL-22 (A) and IL-17 (B) gene expression levels were assessed using quantitative RT-PCR. The messenger RNA values were expressed as mean relative expression ± S.E.M. compared with baseline IL-17 or IL-22 expression from non-colonised individuals after normalizing to β-actin RNA expression (Experimental unit = 1 donor, n = 12/group). Mucosal lining fluid was collected using *Nasosorption*™ *FX·i* devices. IL-22 (C) and IL-17 (D) concentration was measured using a V-plex multiplex ELISA (B). Results are expressed as mean protein expression ± S.E.M (Experimental unit = 1 donor, n=12/group). Statistical analysis was carried out by Mann-Whitney U test.

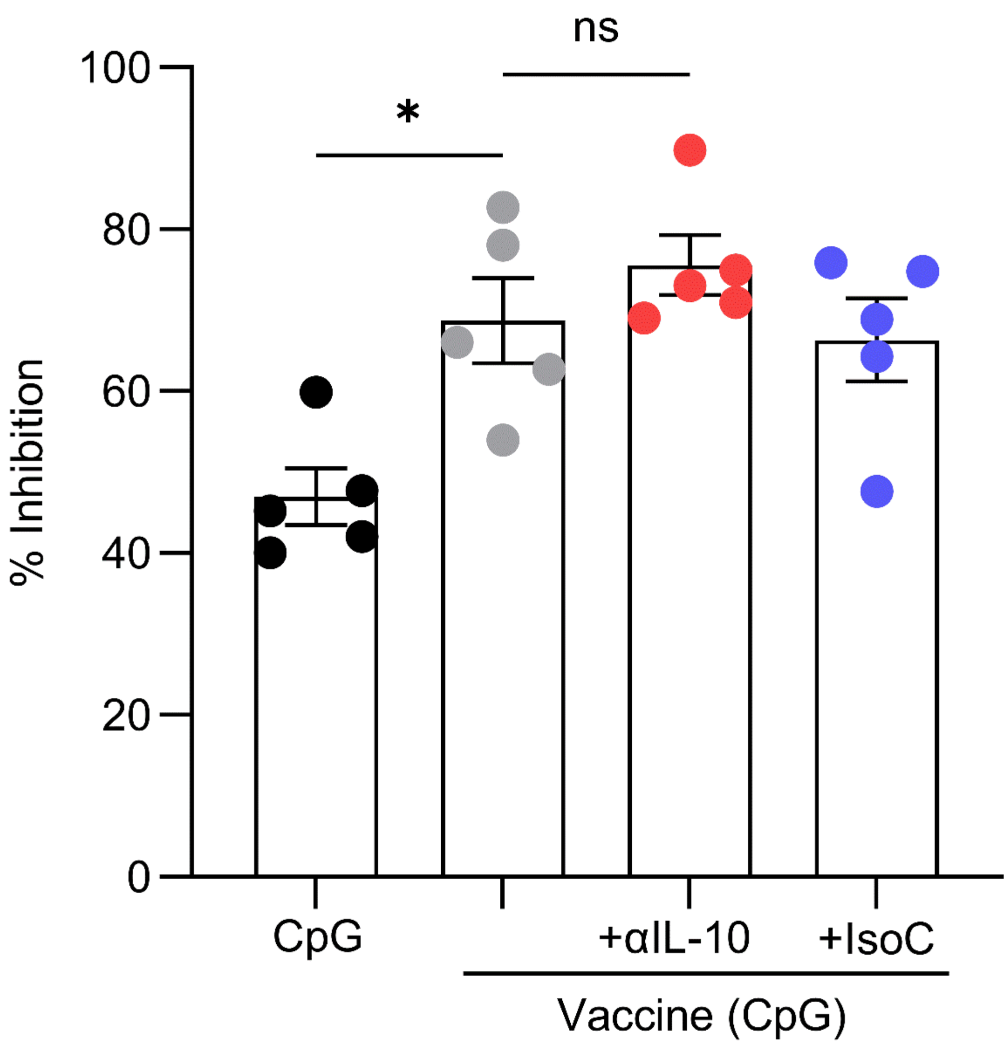

Supplemental Figure 2. Immunisation in the presence of anti-IL10 does not improve humoral immune responses

Mice were immunized with CpG (50µg), vaccine; CpG+ClfA (5µg) or vaccine + anti-IL10 (150µg), or vaccine + isotype control (150µg). All injections were via s.c. injection on day 0, 14 & 28. On day 42 sera was collected from mice. Neutralising antibodies were determined by measuring the ability of the serum of each group to inhibit *S. aureus* adherence via ClfA to fibrinogen. Results are expressed as mean % inhibition ± S.E.M. (Experimental unit = 1 mouse, n =5/group, total # mice used 20, experiment was performed once). Statistical analysis was carried out by one-way ANOVA with Tukey post-test. *P≤0.05

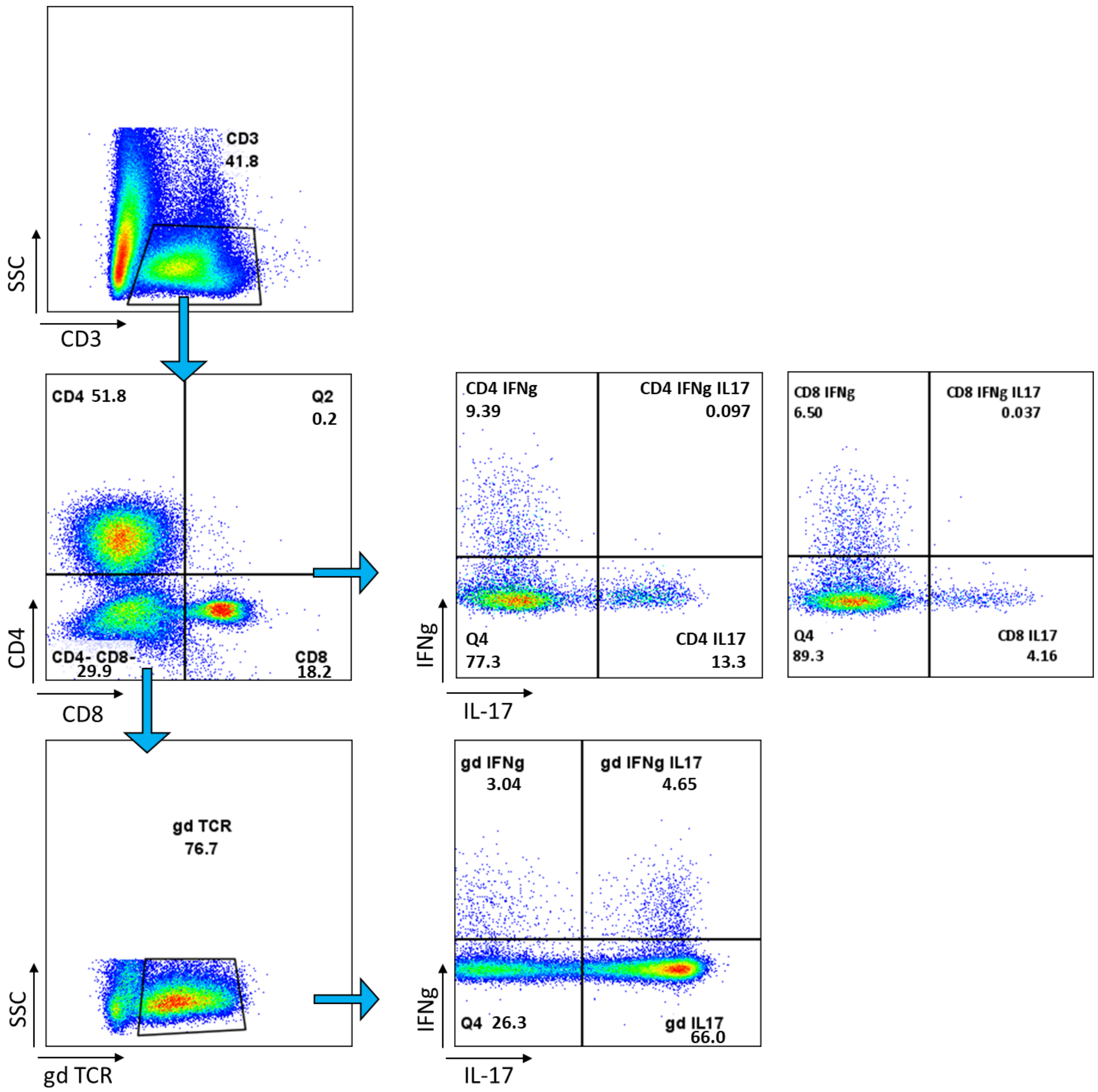

Supplemental Figure 3. Gating strategy for T cell cytokine responses to systemic infection

Mice were immunized with CpG (50µg), vaccine; CpG+ClfA (5µg) or vaccine + anti-IL10 (150µg), or vaccine + isotype control (150µg) via s.c. injection on day 0, 14 & 28. On day 42 mice were challenged with *S. aureus* PS80 (5x10^8 CFU) via i.p. injection. At 24h or 72h post-infection cells of the peritoneal cavity were isolated and gated on CD3+> CD4+/CD8+ or γδ+ T cells. The number of IFNγ+ subtypes, and the number of IL-17+ subtypes in the peritoneum were assessed by flow cytometry. Representative FACS plots are shown depicting the gating strategy used.

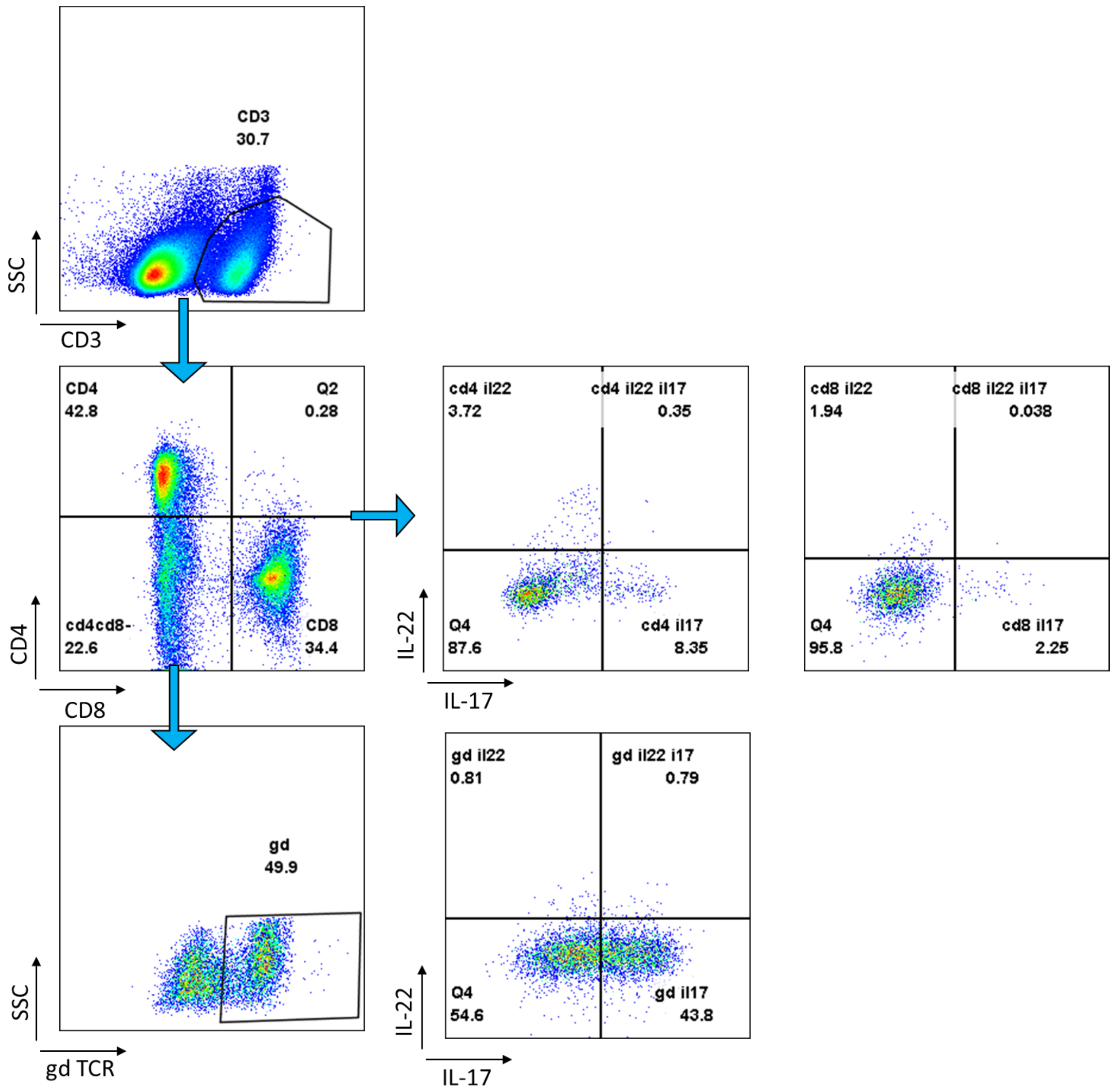

Supplemental Figure 4. Gating strategy for T cell cytokine responses to subcutaneous infection

Mice were immunized with specified adjuvant only, vaccine or vaccine + blocking antibody/isotype control via s.c. injection on day 0, 14 & 28. On day 42 mice were challenged with *S. aureus* USA300 (LAC) (2x107 CFU) via s.c. injection. At 72h post-infection cells of the lesioned skin were isolated and gated on CD3+> CD4+/CD8+ or γδ+ T cells. The number of IL-22+ T cell subtypes, and the number of IL-17+ subtypes in the abscess were assessed by flow cytometry. Representative FACS plots are shown depicting the gating strategy used.

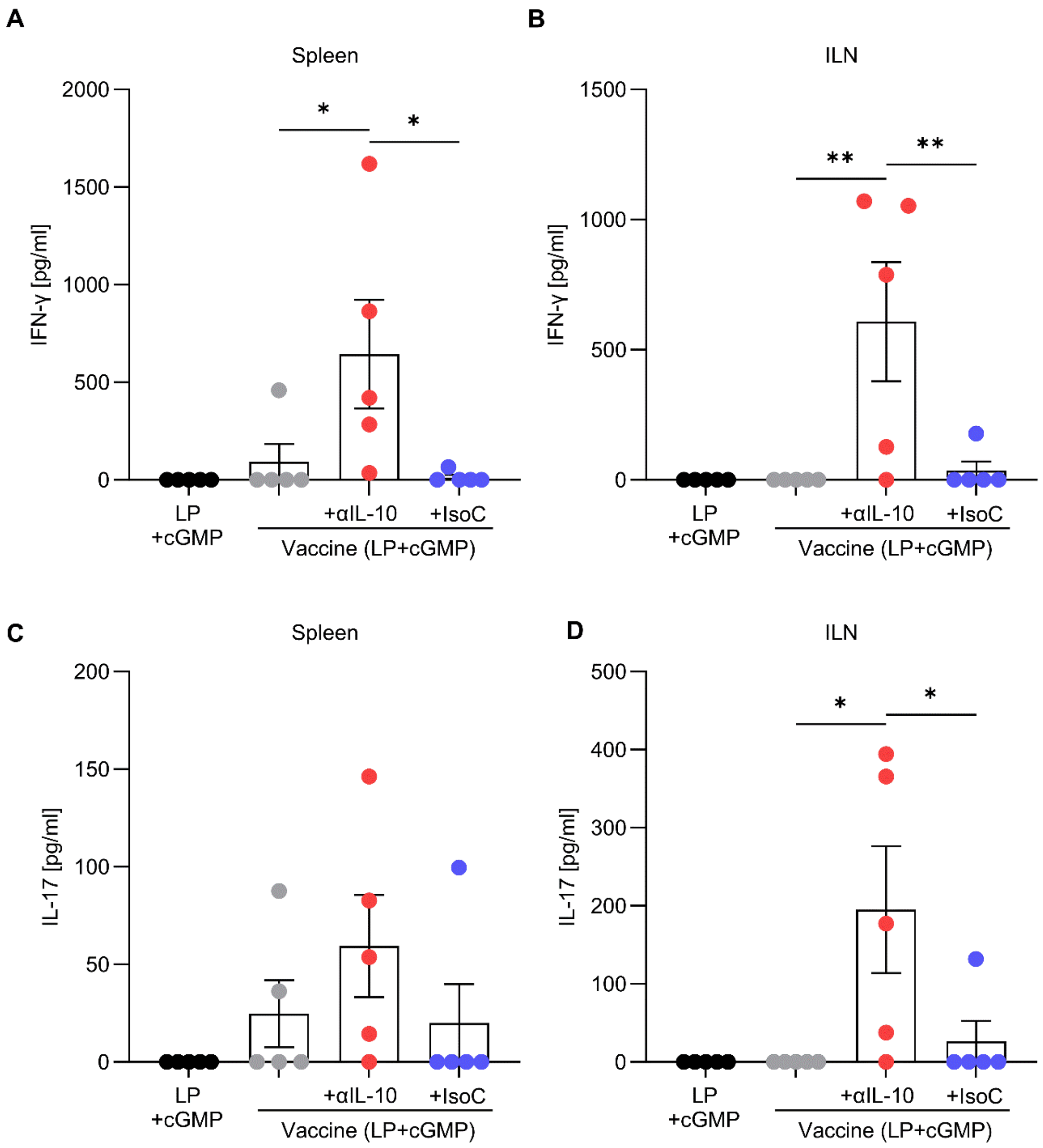

Supplemental Figure 5. Immunisation with an LP+cGMP-based vaccine in the presence of anti-IL10 improves ClfA-specific T cell responses in the spleen and draining ILNs.

Mice were immunized with LP1569 (50µg) + cGMP (10µg), vaccine; LP1569+cGMP +ClfA (5µg) or vaccine + anti-IL10 (150µg), or vaccine + isotype control (150µg). All injections were via s.c. injection on day 0, 14 & 28. On day 42 spleens and inguinal lymph nodes were removed, and ClfA-specific responses were assessed by ex vivo stimulation with media only or ClfA (5µg/ml) for 72h. The levels of IFN-γ (A & B) and IL-17 (C & D) were determined by ELISA. ClfA-specific responses were determined by subtracting responses to media alone. Results are expressed as mean ± S.E.M. (Experimental unit = 1 mouse, n =5/group, total # mice used 20, experiment was performed once). Statistical analysis was carried out by one-way ANOVA with Tukey post-test. **P*≤0.05; ** P≤0.01.

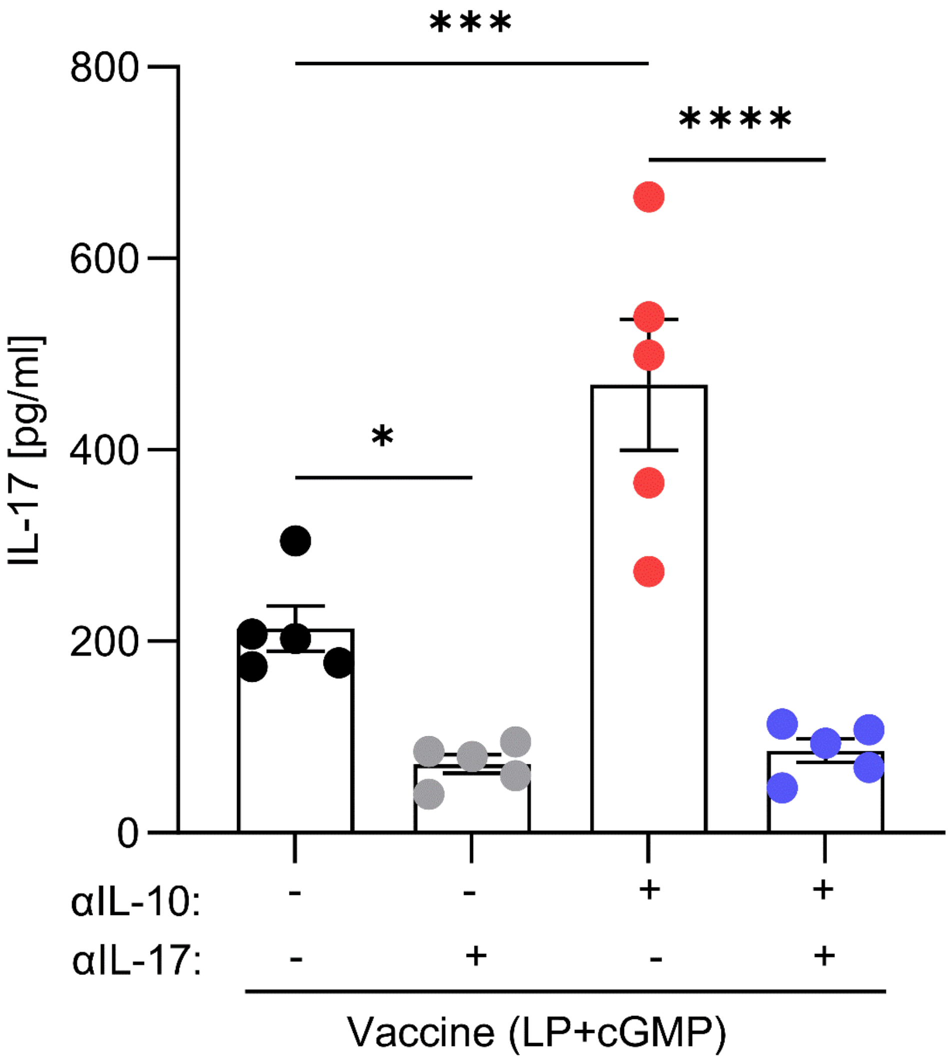

Supplemental Figure 6. IL-17 blocking during subcutaneous S. aureus infection suppresses IL-17 levels in the skin abscess and reduces the protective effect of IL-10 inhibition during vaccination

Mice were immunized with LP1569 (50µg) + cGMP (10µg) +ClfA (5µg) (vaccine) + anti-IL10 (150µg), or vaccine + isotype control (150µg). All injections were via s.c. injection on day 0, 14 & 28. On day 42 mice were subcutaneously administered anti-IL-17 (50µg) or isotype control (50µg) alongside *S. aureus* USA300 (LAC) ($2x10^7$ CFU), and again at 24h post-infection. On day 3 post-infection an 8mm skin punch biopsy was taken at the infection site, homogenized and undiluted homogenate supernatants were then used for IL-17 cytokine production analysis by ELISA. Results are expressed as mean protein expression ± S.E.M (Experimental unit = 1 mouse, n=5/group, total # mice used = 20). Statistical analysis was carried out by one-way ANOVA with Tukey post-test. *P≤0.05, *** P≤0.001, ****P≤0.0001.

Supplemental Table 1. Demographics of human participants

Category	Average Age (years± S.E.M)	Sex	n	% cohort
**Colonised**	31.55 ± 3.26		12	
		Male	5	42
		Female	7	58
**Non-colonised**	32.8± 2.92		12	
		Male	5	42
		Female	7	58

Supplemental Table 2. Primers used in qPCR

Gene	Primer Pair	Supplier
IL10	F: 5’-GCCTTTAATAAGCTCCAAGAG<br>R: 5’-ATCTTCATTGTCATGYAGGC	KiCqStart primers, Sigma Life Science
ACTB *(β-actin)*	F: 5’-GGACTTCGAGCAAGAGATGG<br>R: 5’-AGCACTGTGTTGGCGTACAG	KiCqStart primers, Sigma Life Science
IL22	F:5’-CCTACATGCAGGAGGTGGTG<br>R: 5’-AAACAGCAGGT CAGTTCCC	Integrated DNA Technologies
IL17A	F: 5’-CATTGGTGTCACTGCTACR:<br>R: 5’-TCGGTTGTAGTAATCTGAGG	KiCqStart primers, Sigma Life Science
